# Complementary therapies intervention in Parkinson’s disease: systematic review and meta-analysis

**DOI:** 10.3389/fneur.2025.1703611

**Published:** 2025-11-26

**Authors:** Fabio Sabbadin, Michela Geraci, Laura Nieddu, Daniela D’Imperio, Giorgia Pregnolato, Luisa Cacciante

**Affiliations:** 1 Department of Physical Medicine and Rehabilitation, Venice Hospital, Venice, Italy; 2 Di Maria Fisiosport, Palermo, Italy; 3 SC Neuroriabilitazione e Riabilitazione Funzionale, Sassari, Italy; 4 IRCCS San Camillo Hospital, Venice, Italy; 5 Insight Research Ireland Centre for Data Analytics, University College Dublin, Dublin, Ireland; 6 Laboratory of Healthcare Innovation Technology, IRCCS San Camillo Hospital, Venice, Italy

**Keywords:** complementary therapies, Parkinson disease, art therapy, music therapy, meta-analysis

## Abstract

**Background:**

Parkinson’s disease (PD) is a progressive neurodegenerative disorder characterized by both motor and non-motor impairments, leading to significant declines in quality of life (QoL). While physiotherapy remains a mainstay of non-pharmacological management, interest in complementary therapies—such as music therapy, dance, and art therapy—has grown due to their potential to address motor, cognitive, emotional, and social aspects of PD within a holistic framework.

**Objective:**

To evaluate the effectiveness of complementary therapies compared to usual care (e.g., physiotherapy) in improving balance, functional mobility, freezing of gait (FOG), and QoL in individuals with PD.

**Methods:**

A systematic review and meta-analysis was conducted following PRISMA guidelines and registered in the PROSPERO database (registration code: CRD42025636700). Search was performed on PubMed, Cochrane, Web of Science, and Embase. Eligible studies included randomized or quasi-randomized controlled trials and clinical controlled trials comparing complementary therapies (e.g., music, dance, drama) to usual care in individuals with PD. Primary outcome was balance, whereas functional mobility, FOG, and QoL were set as secondary outcomes. Risk of bias was assessed using the Cochrane RoB2 tool and meta-analyses were performed.

**Results:**

Out of 723 identified records, 29 studies met inclusion criteria and were included for qualitative synthesis. Among these, 17 studies were included in the meta-analysis, whereas risk of bias was performed on 29 studies, revealing scarce methodological quality of the included studies. Balance and functional mobility meta-analyses showed a significant benefit of complementary therapies over usual care.

**Conclusion:**

Complementary therapies demonstrate moderate evidence of benefit in improving balance and functional mobility in individuals with PD, with less conclusive evidence for QoL and FOG. High heterogeneity and methodological limitations across studies highlight the need for more rigorous research.

**Systematic review registration:**

https://www.crd.york.ac.uk/prospero/, CRD42025636700.

## 1 Introduction

Parkinson’s disease (PD) is a progressive neurodegenerative disorder characterized by motor symptoms such as bradykinesia, rigidity, tremor, and postural instability, as well as non-motor symptoms including cognitive decline, sleep disturbances, and autonomic dysfunction (1, 2). Affecting millions worldwide, PD significantly reduces quality of life (QoL) and poses challenges for both patients and caregivers (3–5). While pharmacological and surgical interventions, such as levodopa therapy and deep brain stimulation, remain the gold standard of treatment, their limitations - such as diminishing efficacy over time and adverse side effects - highlight the need for adjunctive strategies (6).

Physiotherapy plays a critical role in the non-pharmacological management of PD. It has been shown to improve mobility, balance, strength, and overall functional independence, addressing key motor impairments. Evidence suggests that regular physiotherapy interventions can mitigate fall risk, enhance gait patterns, and maintain physical fitness, thereby slowing functional decline. Techniques such as balance training, resistance exercises, and gait re-education are core aspects of physiotherapy programs for PD. (7, 8) However, as PD affects more than just motor function, there is an increasing interest in therapies that adopt a more holistic approach, addressing emotional, cognitive, and social well-being alongside physical health (9).

In this context, Complementary Therapies such as art therapy, music therapy, and dance-based interventions have gained prominence (10–12). Music therapy is a clinical discipline involving the purposeful use of music by certified therapists to address cognitive, emotional, social, and physical needs of individuals, often employing techniques such as singing, instrument playing, and songwriting for holistic rehabilitation (13, 14). Another related, but distinct technique, is the use of auditory cueing, a specific rehabilitation tool that uses rhythmic or patterned auditory signals to improve motor timing and gait by facilitating movement synchronization (15, 16). While auditory cueing can be a component within music therapy, it is widely used independently in neurological physiotherapy interventions aimed at motor control enhancement (17, 18). Music therapy has demonstrated benefits in improving gait rhythm, emotional regulation, and cognitive engagement, strengthening the intrinsic connection between auditory stimuli and motor output. Similarly, dance therapy has shown promise in enhancing balance, posture, and movement fluency while fostering social interaction and emotional expression (19–21). Art therapy encompasses a spectrum of activities such as painting, drawing, sculpting, collage, clay modeling, and more, each of which taps into different cognitive and motor skills, and emotional processes, while offering varied levels of interpersonal engagement (22). For example, visual arts primarily stimulate sensory and reflective capacities through solitary creation, while theater therapy involves role-play, dramatic enactment, and social–emotional interaction (23). Art therapy provides a creative outlet for patients, helping to reduce anxiety and depression while promoting a sense of accomplishment and self-expression (24). These therapies not only complement traditional physiotherapy by addressing the broader spectrum of PD symptoms, but also offer engaging and personalized ways for patients to remain active and socially connected (25, 26).

Complementary therapies align closely with the principles of the International Classification of Functioning, Disability and Health (ICF), which emphasizes a holistic view of health that integrates physical, emotional, and social dimensions (27). The ICF framework highlights the importance of addressing not only impairments in body structure and function but also limitations in activities and restrictions in participation (28). Therapies such as music therapy, dance, and art therapy address these domains by fostering motor and cognitive engagement, enhancing emotional well-being, and promoting social interaction (29, 30). By targeting both individual capabilities and contextual factors, these interventions could provide a person-centered approach that supports functional independence and QoL in individuals with PD.

To enable a broader understanding of their potential shared benefits for PD and to reflect their real-world use in holistic care models, the aim of this systematic review is to evaluate the benefits of complementary therapies compared to usual care in the management of PD, with a specific focus on their impact on balance, freezing of gait (FOG) and QoL. Complementary therapies often address overlapping domains, including motor function, emotional well-being, cognitive abilities, and social participation, which are all impacted by PD. (31, 32) By evaluating these therapies together, the study can determine whether their shared characteristics - such as motivation, engagement, and multisensory stimulation - contribute to meaningful improvements over usual care or physiotherapy alone. By analyzing and synthesizing current evidence, this review seeks to determine the relative effectiveness of these interventions in addressing key motor and non-motor challenges faced by individuals with PD, providing insights into their potential role in comprehensive care strategies.

## 2 Methods

### 2.1 Protocol and registration

This systematic review and meta-analysis was registered in PROSPERO database, registration code: CRD42025636700. The review is reported according to the Preferred Reporting Items for Systematic Review and Meta-analysis (PRISMA) (33), and the PRISMA checklist.

### 2.2 Electronic searches

The first search was launched on PubMed, Cochrane Library, Web of Science and Embase databases on 17/01/2023. A second search was performed as an update on 13/08/2024. A detailed description of the search strategy is presented Appendix A.

### 2.3 Eligibility criteria

Our research question was related to the efficacy of complementary therapies on the recovery of balance, the maintenance of motor function, and on QoL, in patients with PD. Accordingly, the eligibility criteria were structured using the PICO (i.e., population, intervention, comparison, and outcome). We applied the following inclusion criteria: patients with PD or Parkinsonism who have received primary treatment based on a complementary therapy described as music therapy, art therapy, drama therapy, or similar. Control groups must have received usual care, such as traditional physiotherapy, home exercises, or drug therapy, otherwise they may not have been treated. We were interested in investigating the effect of these kinds of treatments on balance, (i.e., primary outcome), but also on recovery or maintenance of motor function, on the impairment caused by freezing, and on QoL (i.e., secondary outcomes). Study design included RCT, quasi-RCT, and Controlled Clinical Trial. Searches were limited to English or Italian language without publication’s year limitations. Gray literature was not searched in this review.

### 2.4 Study selection

Database search results were imported into Rayyan, an online free tool used for records screening^1^ and duplicates were removed. For study selection through abstract screening, four reviewers were divided into two groups (i.e., two reviewers for each group). Abstracts that had to be screened were equally divided between the groups. The reviewers, independently, screened records based on title and abstract, using an inclusion/exclusion criteria template. A fifth reviewer was selected to solve any disagreements between reviewers. At the end of this process, full texts of the articles were obtained, and the same procedures were used for full text screening and for the assessment of the methodological quality (i.e., risk of bias assessment). Qualitative and quantitative data were extracted by four reviewers filling synoptic tables created *ad hoc* for this study, which included author’s name, publication date, research setting, study design and study results. Furthermore, a second table was filled with quantitative data used for the meta-analyses.

### 2.5 Outcomes

We aimed to assess the effect of complementary therapies on four outcomes: balance (i.e., primary outcome), recovery or maintenance of motor function, impairment caused by freezing and QoL. Outcome measures included Berg Balance Score (BBS), MiniBESTest, usually used for the assessment of balance in PD, and Timed-Up and Go (TUG) for motor function. For the assessment of the impairment caused by freezing we considered the Freezing of Gait Questionnaire (FOG-Q) and Parkinson’s disease Questionnaire (PDQ-39). Finally, we wanted to evaluate the improvement of perceived-QoL by using questionnaires that assess changes in the perception of QoL (e.g., McGill Quality of Life, FOG-Q and PDQ-39).

### 2.6 Data extraction and risk of bias assessment

The included studies underwent a methodological quality assessment for the risk of bias using the revised Cochrane risk-of-bias tool for randomized trials (RoB2) (34) and the Risk Of Bias In Non-randomized Studies – of Interventions (ROBINS-I) (35). The risk of bias has been assessed at the individual outcome level for the primary outcome (i.e., balance). With the ROB2 tool, we evaluated the following domains: 1- bias arising from the randomization process; 2- bias due to deviations from intended interventions; 3- bias due to missing outcome data; 4- bias in the measurement of the outcome; and 5- bias in the selection of the reported result. For each domain, the judgment on the risk of bias was expressed as “high risk,” in case of a high possibility in the occurrence of bias; “low risk,” in case of a low possibility of bias; “some concern,” when we could not exactly define the real incidence of bias. Regarding non-randomized studies, we assessed methodological quality by analyzing seven bias domains: confounding bias, selection bias, measure intervention bias, performance bias, attrition bias, detection bias, reporting bias. We coded risk of bias for each domain and overall risk of bias as “low risk,” “moderate risk,” “serious risk,” “critical risk” or “no information,” according to the ROBINS-I guidance.

### 2.7 Data synthesis and statistical analysis

We used Review Manager (RevMan) [Computer program] Version 7.12.0.^2^ for statistical analysis (i.e., meta-analysis). Treatment effects were evaluated using mean difference (MD) for homogeneous outcome measures or standardized mean difference (SMD) for the outcomes evaluated with different scales. Confidence interval (CI) for continuous outcomes was identified at 95%. Statistical heterogeneity was assessed with the *I*^2^ statistic, establishing the cut-off value at 50% and considering intervention and outcome measures. We conducted a meta-analysis based on random-effects model or fixed model with 95% CI using RevMan 7.12.0. We explored heterogeneity as detailed above.

## 3 Results

Our search identified 723 results from 4 electronic databases. After removing 214 duplicates, 509 records remained for screening. We excluded 478 records with different populations, interventions or study design than specified in the inclusion criteria and then assessed for eligibility a total of 31 full text articles. After full-text screening, 29 studies remained for qualitative synthesis (i.e., two studies were excluded due to wrong type of publication (36, 37)). Out of these 29 studies, 17 reported data as mean and standard deviations, and were included in the meta-analysis.

**Figure 1:**
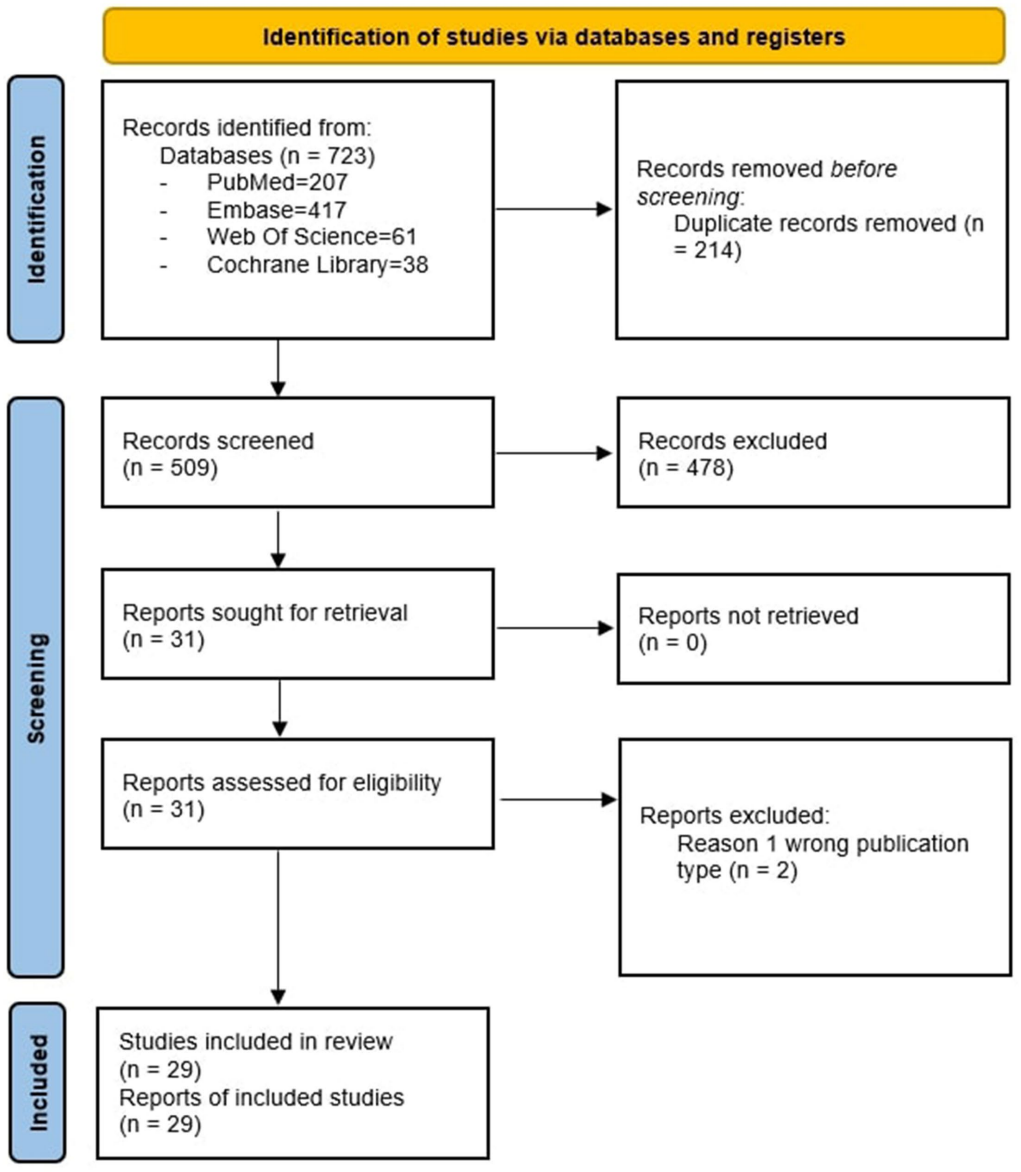
PRISMA flow diagram.

The PRISMA flowchart of the review process is shown in Figure 1.

### 3.1 Characteristics of included studies

The included studies comprised 27 randomized or quasi-randomized controlled trials (38–64), and two non-randomized clinical trial (65, 66).

The interventions included under the umbrella of complementary therapies for PD are heterogeneous but share key features that justify their combined analysis. The main therapies examined include music therapy, dance therapy, and theater therapy, each with distinct yet overlapping mechanisms. *Music therapy* involves both active engagement (singing, instrument playing) and receptive elements (listening), with rhythmic auditory stimulation playing a key role in enhancing motor coordination, cognitive function, and emotional wellbeing (57). Within many interventions, music serves a dual function—as a primary therapeutic modality and as an underpinning tool to facilitate movement and rhythm in other therapies, such as dance and physiotherapy (41). *Dance therapy* integrates music, rhythm, and movement, addressing balance, coordination, posture, and social interaction. Different dance styles stimulate motor learning and emotional expression, leveraging the motivational power of music to enhance adherence and participation (51, 67). *Theater therapy* employs expressive performance and improvisation to stimulate physical activity, communication, and cognitive engagement in a social context, sometimes combining elements of music and movement (52).

Despite their heterogeneity, these therapies converge on common characteristics: multisensory stimulation (auditory, kinesthetic, visual), social engagement, emotional activation, and motivation (68). Music and rhythm are frequently central components that bind many complementary interventions, either as standalone therapies or as tools embedded within others to provide timing cues and enhance motor control (49, 63).

The duration and intensity of the interventions varied substantially across the included studies. Dance-based therapies generally consisted of sessions lasting between 30 and 90 min, conducted once or twice per week, with total intervention periods ranging from 10 weeks to 12 months. In contrast, music-enhanced physiotherapy was typically administered more frequently, often more than twice per week, but over shorter periods, usually between 4 and 8 weeks. The most intensive regimen was observed in the active theater intervention of Modugno and colleagues study, which involved 6-h daily sessions, delivered over two consecutive days, once or twice per month, amounting to approximately 18 h per month, sustained over a 3-year period (52). Overall, the total duration of experimental interventions ranged from 10 to 30 h in most studies, with two studies reaching approximately 100 h of intervention dosage (43, 44), and one study exceeding 600 h overall (52).

Most interventions took place in outpatient or community settings, such as dance studios, rehabilitation centers, or university facilities. Several dance programs (e.g., partnered tango or ballroom classes) were delivered in group formats, often led by professional dancers supported by physiotherapists or researchers. Conversely, music-augmented physiotherapy sessions typically occurred in clinical environments, combining structured motor exercises (e.g., treadmill or gait training) with auditory rhythmic stimuli. Only one program, in addition to the dance classes, included a home-based dance component to be performed three times per week, guided by a video provided on DVD or CD-ROM (58).

In relation to outcomes assessed, balance was evaluated in 17 studies. Out of these, 10 studies primarily used BBS (42, 45, 46, 48, 50, 51, 59, 60, 62, 63), whereas 7 used the Mini-BESTest (43, 44, 54–56, 63, 64). Other tools included Falls Efficacy Scale (FES) (54, 62, 63), the Fullerton Advanced Balance (FAB) (42, 46, 50), and the Activities-specific Balance Confidence (ABC) scale used in 2 studies (48, 63).

A total of 26 studies assessed participants’ functional mobility. The most used tool was the TUG, reported in 18 studies (41, 42, 44–46, 48, 50, 51, 54, 56, 57, 59, 60, 62–66), followed by 6 Minute Walking Test (6MWT) in 7 studies (42–44, 58, 59, 63, 64), the 10 Meter Walking Test (10MWT) in 3 studies (41, 62, 63), Gait Deviation Index (GDI) in 2 studies (42, 57), Functional Gait Assessment (FGA) (62) and Dynamic Gait Index (DGI) (64).

QoL was investigated in 13 studies, with the PDQ-39 being the most frequently used outcome measure (38, 50–52, 54–58, 60, 66), followed by PDQL in 1 study (53), and the McGill Quality of Life Questionnaire in 1 study (64).

Finally, FOG was measured in 7 studies using the FOG-Q (45, 48–50, 54, 56, 60). Qualitative details of the included studies are reported in the Supplementary Table 1S.

### 3.2 Excluded studies

After full-text screening, we excluded 2 studies as one was a letter to the editor and the other a short communication, which limited the availability of relevant data for inclusion (36, 37).

### 3.3 Risk of bias in randomized studies

**Figure 2:**
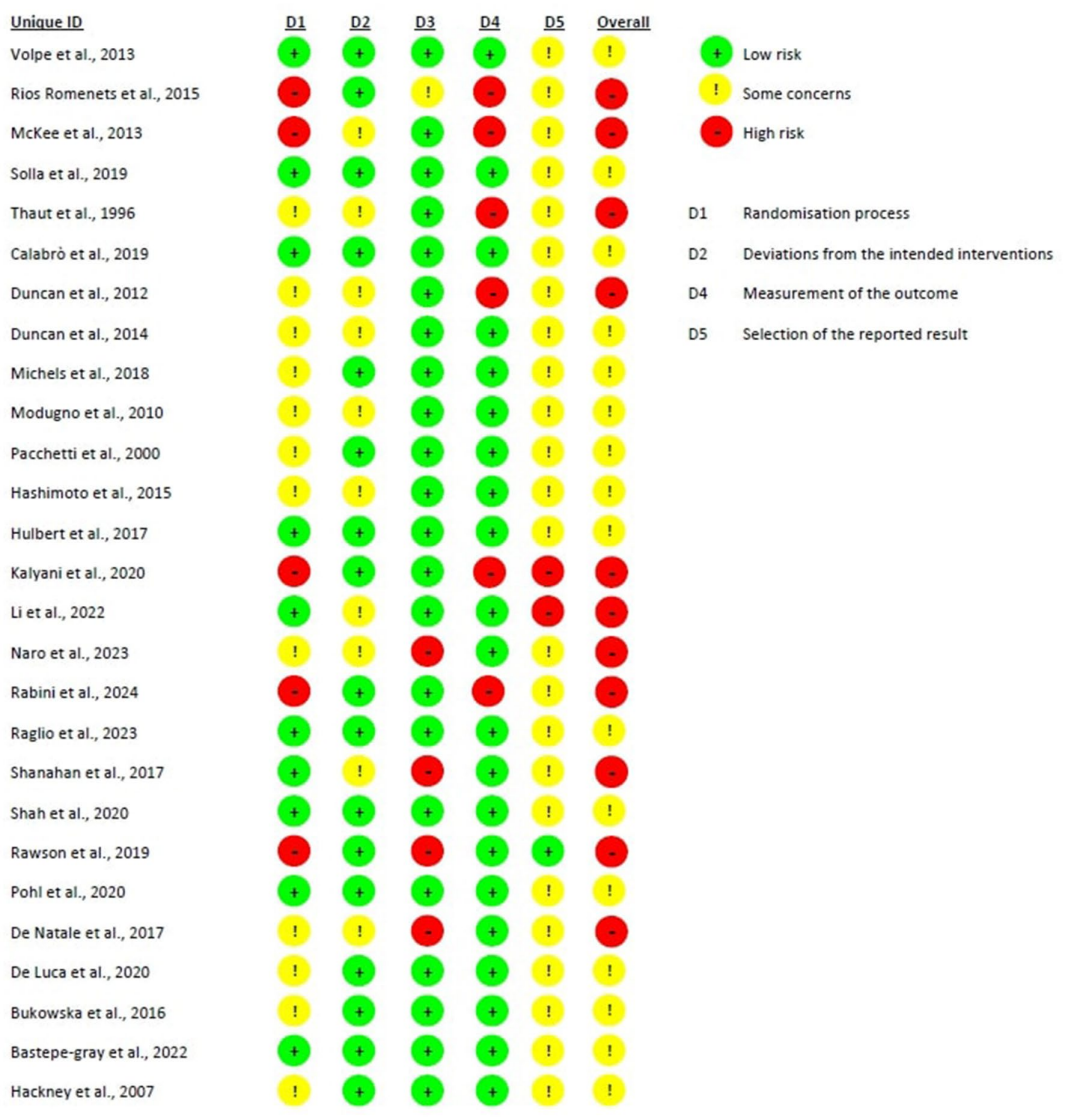
Risk of bias of randomized studies.

Figure 2 shows the risk of bias in the included studies

- Bias arising from the randomization process: 10 studies were assessed with a low risk of bias, as the authors described a correct randomization process and there were no differences between intervention groups related to this process (38, 40, 47, 49, 54, 57–60, 64). Five studies were judged with a high risk of bias, as the participants were randomized according to clinical needs or a randomization procedure was not carried out (48, 50, 55, 56, 63). Twelve studies were judged with some concerns regarding the risk of bias, as no information was provided (39, 41–46, 51–53, 61, 62).
- Bias due to deviations from the intended interventions: Seventeen studies had a low risk of bias in this domain (38–41, 45, 47, 48, 51, 53–57, 59, 60, 63, 64). In the remaining 10 studies, not enough information were reported, leading to some concerns regarding the risk of bias.
- Bias due to missing outcome data: all the studies had a low risk of bias in this domain except for 5 studies. Four of them had a high risk of bias because several patients dropped out (40 and 28%) and no evidence were provided on missing data handing (42, 55, 58, 62). The remaining one study had a high dropout rate (over 20%) however, the reasons for the dropouts were explained, and the outcomes did not appear to be influenced by the underlying values being studied, leading to a judgment of some concerns regarding the likelihood of the presence of bias (56).
- Bias in measurement of the outcome: Twenty-one studies were judged to have a low risk of bias in this domain. However, 6 studies were assessed as having a high risk of bias due to the use of outcome measurement methods that appeared suboptimal and, in all cases, the assessors were not blinded to group allocation, which may have influenced the outcome assessment (43, 48, 50, 56, 61, 63). Indeed, the latter was performed through scales that can be influenced by knowledge of the intervention received (e.g., BBS, MiniBesTest).
- Bias in the selection of the reported result: only one study was judged with a low risk of bias, as data were in accordance with a pre-specified analysis plan and with the protocol provided (55). Two studies had a high risk of bias because the reported results were not in accordance with the study protocol (48, 49), whereas the remaining 24 studies were judged with some concerns for this domain as there were no information about the study protocol nor about an analysis plan.

Overall bias: 16 were judged as having some concerns regarding the likelihood of the presence of bias, mainly due to the absence of a study protocol or an analysis plan, raising therefore some concerns in relation to the selection of the reported results (38–41, 44–47, 51–54, 57, 59, 60, 64). The remaining 11 studies were judged to have an overall high risk of bias, mainly due to lack in the randomization process or in outcome measurements.

### 3.4 Risk of bias in non-randomized studies

Supplementary Table 2S provides a comprehensive assessment of risk of bias in the included non-randomized studies. The following risks were identified:

- Confounding bias: one study demonstrated low risk of bias by adequately measuring and controlling for potential confounders (65). In contrast, the other study had a serious risk of bias as all known important confounding domains were neither measured nor adjusted for (66).
- Selection bias: one study showed low risk for selection bias in participant inclusion (65), while the other had a moderate risk due to uncertainty regarding whether the initiation of follow-up coincided with the start of the intervention (66).
- Measure intervention bias: both studies were judged to have a moderate risk of bias in the classification of intervention status because of insufficient reporting on the assignment procedures.
- Performance bias: both trials were assessed as having low risk for deviations from intended interventions, since no departures from standard practice were detected.
- Attrition bias: low risk was found for both studies, as outcome data were fully reported.
- Detection bias: both studies were judged with a low risk of detection bias, as methods of outcome assessment were comparable across intervention groups and outcome measures could not have been influenced by knowledge of the intervention received.
- Reporting bias: for the selection of the reported results, the study of Dos Santos Delabary et al. were judged with a serious risk, as PDQ-39 and UPDRS III were specified in the protocol but not reported in the study (65), whereas Ventura et al. did not report any information of the presence of a protocol nor a statistical analysis plan (66).

Overall, both studies were assessed with a serious risk of bias, mainly due to issues related to the selection of the reported results (65) and possible unaddressed confoundings (66).

### 3.5 Effects of intervention

#### 3.5.1 Comparison 1. Complementary therapies versus usual care, outcome: 1.1 balance

**Figure 3:**
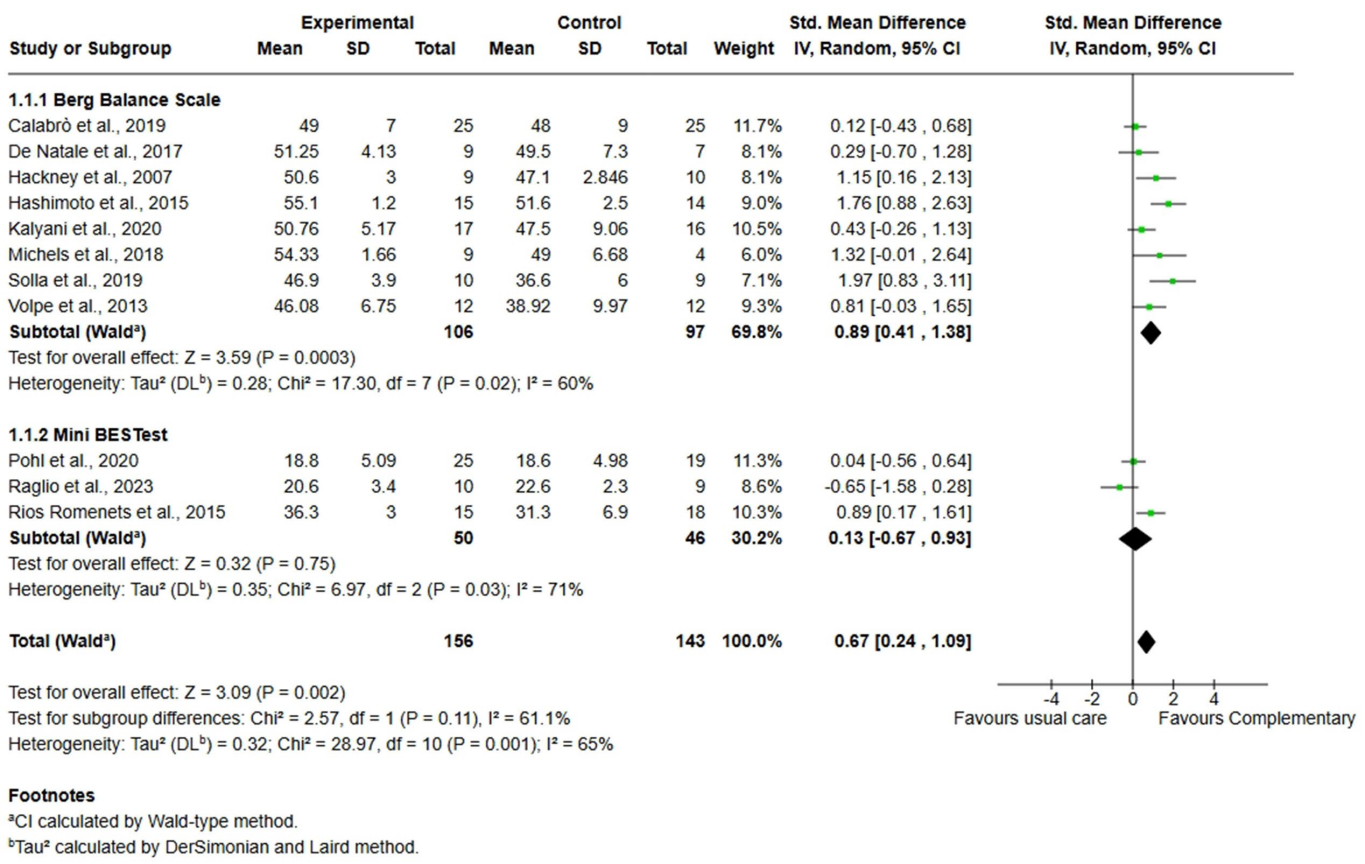
Comparison 1. Complementary therapies versus usual care, outcome: 1.1 balance.

Eleven studies, with 299 participants overall (156 in the experimental group and 143 in the control group) were analyzed for balance, through analysis of the results from BBS and MiniBESTest. The analysis was performed using SMD with random effect model and confidence interval (CI) of 95%. The meta-analysis showed a significant difference between the two treatment modalities in favor of complementary therapies, overall (SMD = 0,67; 95% CI 0.24, 1.09, *I*^2^ = 65%). When looking at the subgroup analysis we performed based on assessment tools (i.e., BBS and MiniBESTest), the meta-analysis showed a significant difference in favor of complementary therapies (SMD = 0.89; 95% CI 0.41,1.38, *I*^2^ = 60%) when balance is assessed with BBS. Conversely, no differences were found when MiniBESTest was analyzed (SMD = 0.13; 95% CI -0.67, 0.93, *I*^2^ = 71%) (Figure 3).

#### 3.5.2 Comparison 1. Complementary therapies versus usual care, outcome: 1.2 functional mobility

**Figure 4:**
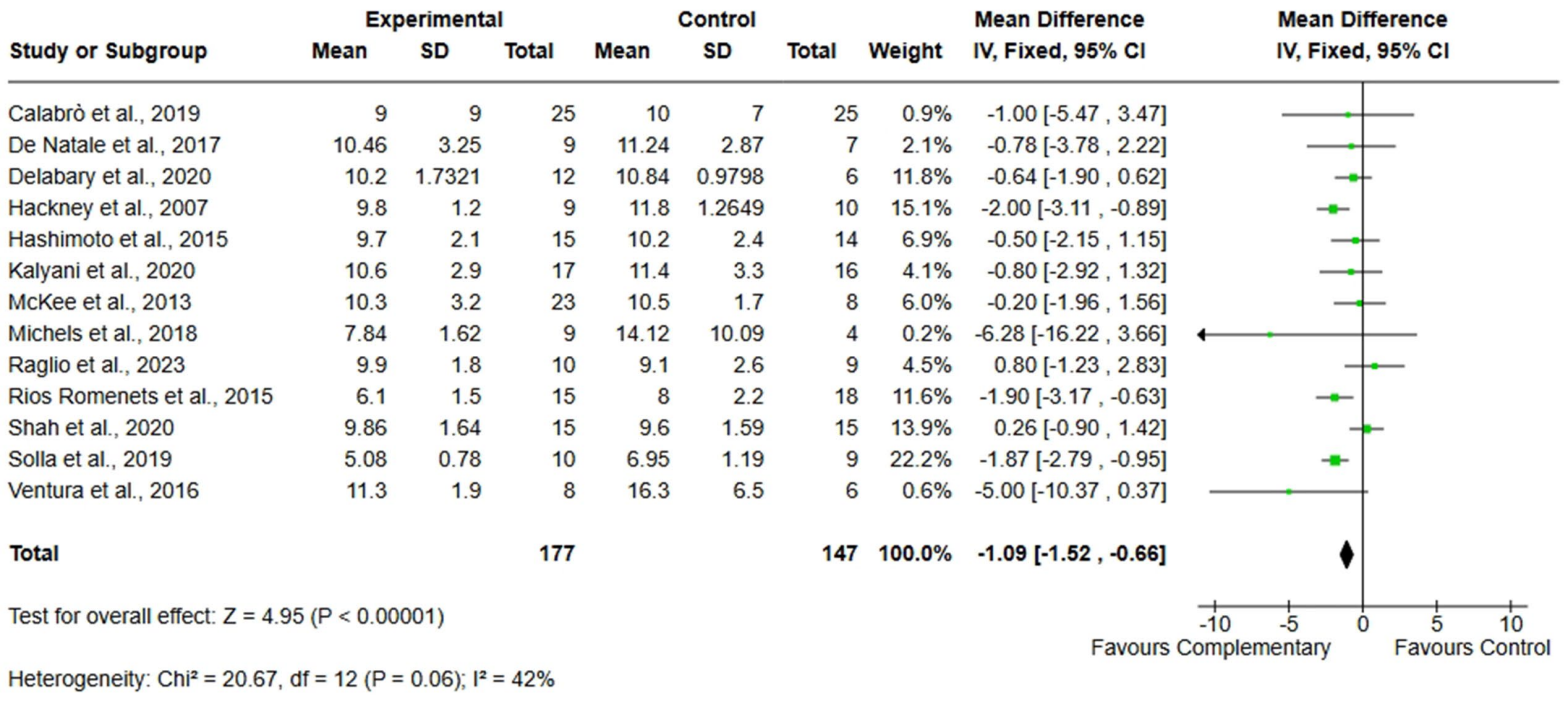
Comparison 1. Complementary therapies versus usual care, outcome: 1.2 functional mobility.

A total of 13 studies, including 324 participants (177 in the experimental group and 147 in the control group), were analyzed to evaluate improvement in functional mobility using the TUG test. The analyses were performed using MD with fixed effect model. The meta-analysis showed a statistically significant improvement in the experimental group compared to usual care (MD = −1.09; 95% CI: −1.52 to −0.66; *I*^2^ = 42%) (Figure 4).

#### 3.5.3 Comparison 1. Complementary therapies versus usual care, outcome: 1.3 freezing of gait

**Figure 5:**
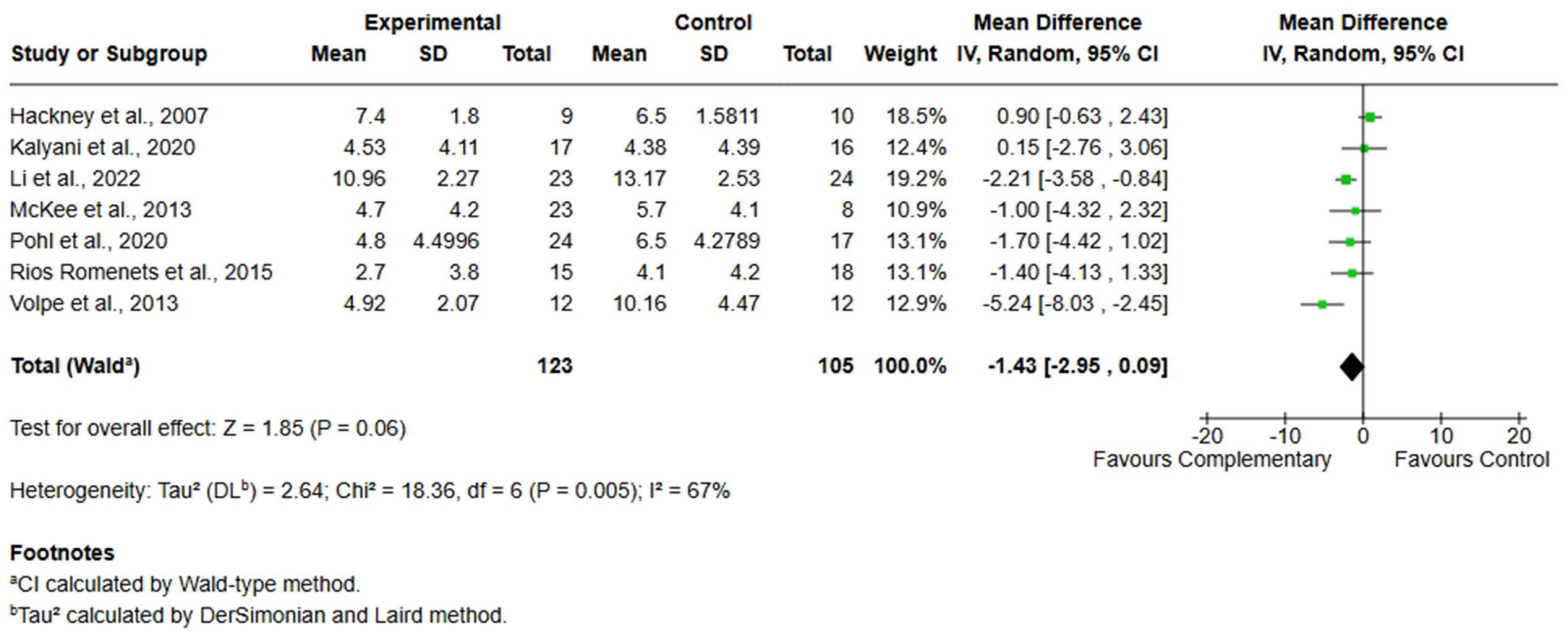
Comparison 1. Complementary therapies versus usual care, outcome: 1.3 freezing of gait.

Seven studies investigated the impact of FOG on mobility and QoL by administering the FOG-Q to a total of 228 participants (123 in the experimental group and 105 in the control group). The meta-analysis, performed using MD and random-effects model, revealed a slight difference in favor of complementary therapies compared to usual care (MD = −1.43; 95% CI -2.95, 0.09, *I*^2^ = 67%); however, the effect was not statistically significant in improving FOG (Figure 5).

#### 3.5.4 Comparison 1. Complementary therapies versus usual care, outcome: 1.4 quality of life

**Figure 6:**
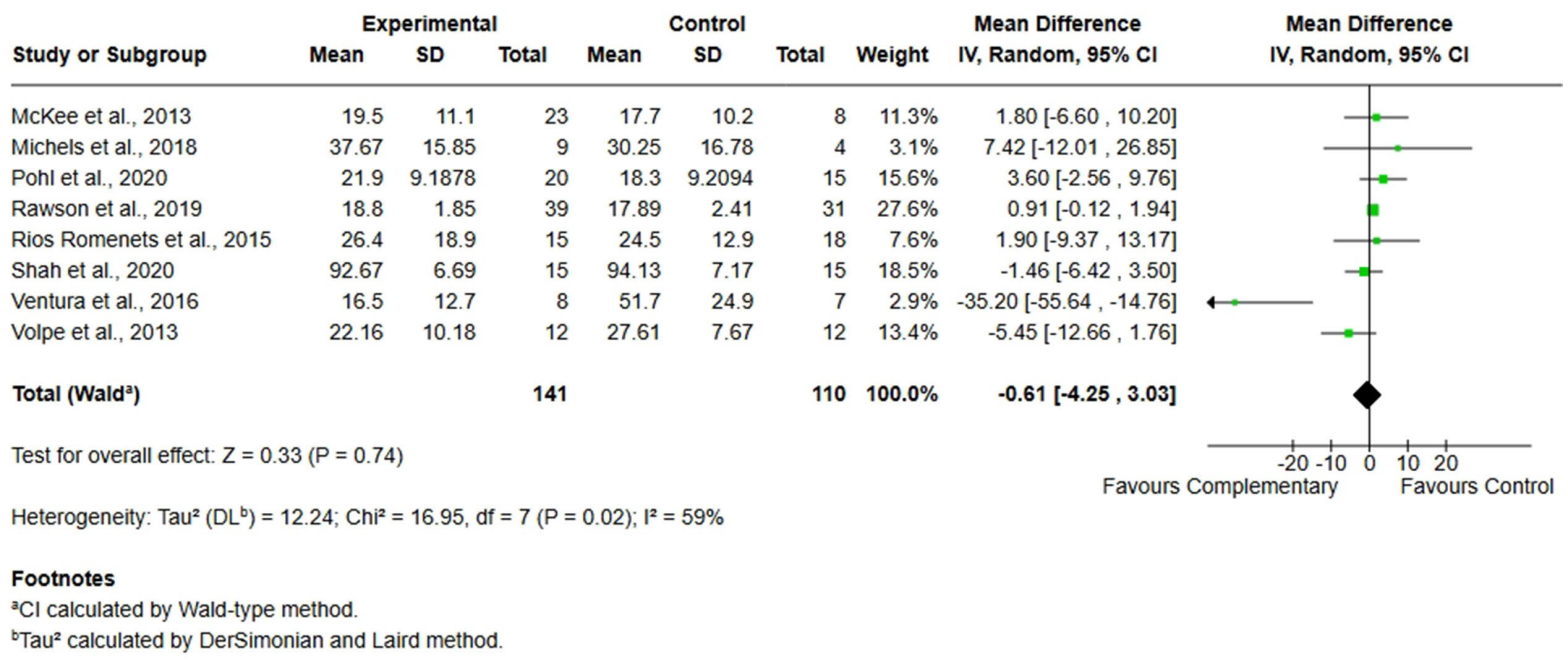
Comparison 1. Complementary therapies versus usual care, outcome: 1.4 quality of life.

Seven studies, comprising 251 participants (141 in the experimental group and 110 in the control group) were included in the analysis of QoL. The studies assessed this domain with the PDQ-39 questionnaire. Also in this case, analysis was performed using MD with random effects model, and no significant differences were found between complementary therapies and usual care (MD = −0.61 95% CI -4.25, 3.03, *I*^2^ = 59%) (Figure 6).

## 4 Discussion

Complementary therapies in PD encompass distinct but often overlapping modalities including dance, drama, and music-based interventions (14). Movement-based therapies (e.g., dance) focus on rhythmic motor activity, balance, and coordination, while drama-based interventions foster expressive communication and social engagement (68). Music-based therapies target both motor and non-motor domains through rhythmic auditory stimulation and structured musical activities (69). Although these modalities can be described separately based on their primary mechanisms, in practice they frequently overlap, as music is integral to both dance and drama (68). What distinguishes complementary therapies from clinical tools such as music cueing is the holistic, person-centered focus: complementary therapies are designed to address the emotional, cognitive, physical, and social needs of patients, operated by trained professionals, whereas clinical tools are adjunct techniques aimed at facilitating specific rehabilitation goals. A clear example of the adjunctive features of complementary therapies that can offer greater benefits is represented by theater therapy. In this context, theater training represents a very effective form of emotional/holistic rehabilitation because, due to its similarity to real-life situations, patients might learn or relearn social and emotional strategies in a protected environment and transfer them to everyday life situations (23, 70). As supported by recent systematic reviews and mechanistic studies, this holistic approach delivers broad and meaningful benefits for individuals with PD. (14, 68)

The results of the meta-analysis on balance suggest that complementary therapies may offer greater benefits than usual care in improving postural stability in individuals with PD. Nevertheless, this conclusion should be interpreted with caution, as the analysis revealed a moderate level of heterogeneity. This heterogeneity could derive from several factors, including variations in how experimental interventions were delivered, differences in the types of complementary therapies applied across studies, the methodological quality of the studies and the variability in follow-up time points across studies. The impact of heterogeneity becomes particularly evident in the subgroup analyses. Specifically, among the studies using the Mini-BESTest as the primary outcome measure, heterogeneity increased substantially (*I*^2^ = 71%), reflecting greater discrepancies in the results. Notably, one study reported findings that favored usual care over complementary intervention, highlighting again the conflicting results on this topic (56). It has to be said that the use of the BBS in several studies included in the review raises concerns regarding its sensitivity in detecting balance impairments specific to PD. While it remains a widely used and validated tool for general balance assessment, the BBS may not adequately capture dynamic postural control, anticipatory adjustments, or balance during gait - domains particularly affected in PD patients, and present ceiling effect (71). In this context, the Mini-BESTest would represent a more appropriate choice, as it is specifically designed to assess balance deficits commonly observed in PD and has demonstrated greater sensitivity to detect changes following intervention (72).

Taken together, while the overall trend appears promising, the observed variability across studies and the presence of conflicting results makes it difficult to confirm the reliability and generalizability of the conclusions regarding balance outcomes.

A similarly cautious interpretation is warranted for the results concerning functional mobility. Although the same sources of heterogeneity apply - such as differences in intervention protocols and in complementary approaches - the meta-analysis in this domain showed a lower heterogeneity index (*I*^2^ < 50%) and a statistically significant effect. This suggests that experimental interventions may have a more consistent impact on improving motor performance compared to usual care as reported in other systematic review with meta-analysis where they investigated specifically types of complementary therapies and have shown improvements in function mobility (26, 73–75). Even if the mechanisms through which individuals with PD benefit from dance and other art-based interventions remain the subject of ongoing investigation, approaches such as dance, music-based movement, and theatrical activities integrate physical, cognitive, and emotional components that may synergistically enhance motor function, cognitive processing, and psychological well-being (68, 76). Furthermore, it is plausible that auditory stimuli provide an “added value” within the rehabilitation context by increasing the motivational and affective salience of motor tasks, thereby improving adherence and potentially amplifying the functional outcomes when compared to movement training alone (75).

In contrast, the meta-analysis of QoL demonstrated high heterogeneity and a lack of statistically significant effects, indicating no clear advantage of complementary therapies over usual care in this domain. However, sensitivity analysis revealed that the exclusion of a single study (66) would reduce heterogeneity to zero (*I*^2^ = 0%). Despite this reduction, the overall effect size continued to overlap with the line of no effect, reinforcing the uncertainty around the comparative efficacy of the interventions on QoL, in accordance with the results of other studies (26, 73, 74, 77, 78).

A comparable scenario emerged for the outcome of FOG. The meta-analysis did not yield statistically significant results in favor of either complementary therapies or usual care. Moreover, the analysis was affected by a high degree of heterogeneity, which further complicates the interpretation and generalizability of the findings. These inconsistencies may be reflected by the limited number of studies addressing FOG, as well as the variability in intervention types, assessment tools and in treatment dosage, as already pointed out previously. Currently, scientific literature does not provide clear or consistent evidence supporting the effectiveness of complementary therapies in improving FOG in individuals with PD (26, 77, 79). Further high-quality, targeted research is needed to explore whether specific therapeutic components or delivery methods might be more effective in addressing this particularly debilitating motor symptom.

### 4.1 Implications for research and practice

To advance the field and support future research, efforts are currently being put on to develop theoretical frameworks capable of explaining and standardizing the multifaceted nature of these interventions (77). These models seek to clarify how specific elements - such as rhythmic auditory stimulation, structured movement, emotional engagement, and social interaction - integrate to produce therapeutic effects across neurological, physical, psychological, and social domains. A clearer conceptual framework would not only improve understanding of how these therapies work, but also guide the development of more targeted and effective interventions, enhance comparability across studies, and strengthen their integration into clinical practice. Further investigation into these mechanisms is essential, not only to enhance the understanding of complementary therapies itself, but also to extend these insights to other forms of art-based and complementary therapies, such as singing (11) or Tai Chi (80), which have shown preliminary evidence of benefit for individuals with PD. By identifying the core therapeutic elements that are common across these interventions, it may become possible to tailor treatment strategies to individual preferences and characteristics. This person-centered approach could improve adherence, optimize outcomes, and ensure that each patient receives the complementary therapy that best aligns with their personality, motivations, and needs, which are all aspects that need to be taken into consideration when rehabilitating individuals with PD as they could concur to a better performance (81). This deeper understanding would not only enable the development of more targeted and personalized interventions but could also inform the design of preventive programs to be implemented in the early stages of the disease, as well as community-based health promotion initiatives. Such programs could be delivered through patient associations or local organizations, thereby facilitating wider access to services, promoting patient empowerment, and potentially reducing the burden on national healthcare systems (82, 83).

## 5 Limitations

In relation to the methodological quality of the studies, many of the included studies presented issues related to participant’s randomization, lack of blinding of assessors or participants, and in relation to the availability of the study protocol or of a pre-specified analysis plan, all of which pose significant challenges to the internal validity of the findings. Furthermore, a key limitation of this review lies on the high heterogeneity observed across the interventions included. On one hand, it is particularly related to their frequency and duration; on the other hand, it depends on the type of interventions themselves, as they are different in nature. Indeed, they encompass dance, drama, and music, each with unique mechanisms, components, and clinical applications. This variability represents a core methodological challenge typical in reviews of complementary therapies (84), and complicates the interpretation of pooled results, limiting the ability to make generalizable conclusions about the effectiveness of complementary therapies in PD.

## 6 Conclusion

Complementary therapies may provide meaningful benefits for individuals with PD, particularly in the domains of balance and functional mobility. However, these findings must be interpreted with caution due to the moderate to high heterogeneity across studies, variability in intervention types and protocols, and methodological limitations. Evidence for QoL and FOG remains inconclusive, with no statistically significant effects and substantial variability across studies. These results underscore the need for high-quality, targeted trials that adopt sensitive and standardized assessment tools, clearer intervention frameworks, and longer follow-up periods. Future research should focus on identifying the core therapeutic elements shared across interventions, optimizing protocols for individual needs, and exploring their potential role not only in rehabilitation but also in preventive and community-based health promotion programs.

## Data Availability

The original contributions presented in the study are included in the article/Supplementary material, further inquiries can be directed to the corresponding author/s.

## Footnotes

1
https://rayyan.qcri.org

2 Available at revman.cochrane.org
