# Supplementary material for: Complementary therapies intervention in Parkinson’s disease: systematic review and meta-analysis
Source: Front Neurol. 2025 Nov 26;16:1703611. doi: 10.3389/fneur.2025.1703611 (PMC12689419; doi:10.3389/fneur.2025.1703611)
Supplement: Supplementary file 1 [file Data_Sheet_1.docx]

***Supplementary Material***

# Appendix A – search strategy

**PUBMED**

#1 ("Parkinson Disease"[Mesh] OR "Parkinsonian Disorders"[Mesh] OR Parkinson* OR “Parkinson Disease” OR “Parkinsonian Disorder*” OR parkinsonism)

AND

#2 (“Drama Therap*” OR “Art Therap*” OR “Dance therap*” OR “Music Therap*” OR “Tango Danc*” OR Tango OR “Argentine Tango” OR “Irish Danc*” OR “Therapeutic Danc*”)

AND

#4 “Function* Recover*" OR Balance OR gait OR “walking speed” OR freezing OR “motor function*” OR “upper limb function*” OR “lower limb function*” OR "Motor function maintenance" OR ADL OR "Activities Of Daily Living” OR “Quality of life” OR QOL

**EMBASE**

#1 ("Parkinson Disease"/de OR "Parkinsonian Disorders"/de OR Parkinson* OR “Parkinson Disease” OR “Parkinsonian Disorder*” OR parkinsonism)

AND

#2 (“Drama Therap*” OR “Art Therap*” OR “Dance therap*” OR “Music Therap*” OR “Tango Danc*” OR Tango OR “Argentine Tango” OR “Irish Danc*” OR “Therapeutic Danc*”)

AND

#4 (“Function* Recover*" OR Balance OR gait OR “walking speed” OR freezing OR “motor function*” OR “upper limb function*” OR “lower limb function*” OR "Motor function maintenance" OR ADL OR "Activities Of Daily Living” OR “Quality of life” OR QOL)

**WEB OF SCIENCE**

WC=(Rehabilitation)

#1 TS=(Parkinson* OR “Parkinson Disease” OR “Parkinsonian Disorder*” OR parkinsonism)

AND

#2 TS=(“Drama Therap*” OR “Art Therap*” OR “Dance therap*” OR “Music Therap*” OR “Tango Danc*” OR Tango OR “Argentine Tango” OR “Irish Danc*” OR “Therapeutic Danc*”)

AND

#4 TS=(“Function* Recover*" OR Balance OR gait OR “walking speed” OR freezing OR “motor function*” OR “upper limb function*” OR “lower limb function*” OR "Motor function maintenance" OR ADL OR "Activities Of Daily Living” OR “Quality of life” OR QOL)

**COCHRANE**

#1 MeSH descriptor: [Parkinson Disease] explode all trees

#2 MeSH descriptor: [Parkinsonian Disorders] explode all trees

#3 ((Parkinson* OR “Parkinson Disease” OR “Parkinsonian Disorder*” OR parkinsonism)):ti,ab,kw

#4 (#1 OR #2 OR #3)

#5 ((“Drama Therap*” OR “Art Therap*” OR “Dance therap*” OR “Music Therap*” OR “Tango Danc*” OR Tango OR “Argentine Tango” OR “Irish Danc*” OR “Therapeutic Danc*”)):ti,ab,kw

#6 ((“Function* Recover*" OR Balance OR gait OR “walking speed” OR freezing OR “motor function*” OR “upper limb function*” OR “lower limb function*” OR "Motor function maintenance" OR ADL OR "Activities Of Daily Living” OR “Quality of life” OR QOL)):ti,ab,kw

# #7 (#4 AND #5 AND #6)

Table 1S. Characteristics of included studies.

| **Author (year)** | **Groups characteristics** | **n** | **Intervention dose** | **Type of therapy** | **Outcome measures** | **Conclusions** |
| --- | --- | --- | --- | --- | --- | --- |
| Bastepe-Gray et al. (2022)^1^ | (1) PD + guitar intervention (EG)  (2) PD + usual treatment (CG)  All subjects in Hoehn and Yahr Stages 2–4 | (1) 12  (2) 12 | Total trial duration was 12  weeks, with a cross-over point at 6 weeks.  Experimental condition consisted of 1hour of guitar group class (2times x week) in addition to usual treatment. | Control condition was usual treatment.  Experimental condition was fingerstyle guitar group class. The intervention was  implemented by professional guitar pedagogues in a community  setting at a community music school. The lesson was designed to include music that promoted finger isolation, reach and grab velocity, and eye-hand oordination  timing and accuracy. Classical guitars were used. | BDI-II, PDQ-39, MDS-UPDRS, typing test (n. of correct sequence in 30 seconds), PPT, BBT, Q-DASH, AES-C; satisfaction questionnaire, Edinburgh Handedness Inventory; Waterloo Handedness Questionnaire-Revised | Group guitar classes can be a feasible intervention in PD and may improve mood, anxiety, and quality of life.  The quality of life improvement was reduced 6 weeks after completion of guitar classes, suggesting that ongoing exposure to intervention is necessary to sustain improvements. |
| Bukowska et al. (2016)^2^ | (1) PD + NMT (EG)  (2) PD + no treatment (CG)  All subjects in Hoehn and Yahr Stages 2–3 | (1) 30  (2) 25 | 4 times a week, for 4 weeks. Every session duration: 45-min. | EG: TIMP, PSE and RAS were used in every 45-min session. Movements similar to those performed in daily life were simulated through PSE, TIMP, RAS.  CG: participants were asket to maintain their daily life activities (walking, stairs) | Spatiotemporal gait parameters, with eyes open and closed (Gait Analysis – Optoelectrical 3D Movement Analysis, BTS System): stance phase, swing phase, double support, stride time, cadence. | NMT sensorimotor techniques  may be employed to improve gait and other rhythmical activities for PD patients. Changes in stability without visual control indicate improvement of proprioception, giving a new compensatory strategy for movement and postural control through auditory system. |
| De Luca et al. (2020)^3^ | (1) PD + treadmill integrated with music therapy (EG)  (2) PD + traditional gait training (CG) | (1) 20  (2) 20 | 3 times a week for 8 weeks, for a total of 24 sessions, each lasting about 30 min. | CG = traditional over ground gait training  EG = gait training with Biodex Gait Trainer, a platform that integrates gait training via a treadmill and RAS.  Both groups performed also traditional PT by means of exercises aimed at improving postural stability, lower limb joint mobilization, muscle  stretching and motor coordination. | PGWBI; Brief-COPE; FIM; TUG; 10mWT | Music assisted gait training may be a complementary  and valuable tool in improving motor symptoms, behavioral, psychological status, and QoL in PD patients. |
| De Natale et al. (2017)^4^ | (1) PD + DT (EG)  (2) PD + TR (CG) | (1) 9  (2) 7 | Both groups attended  plenary classes of 20 sessions lasting 60 minutes  each, twice weekly, for 10 weeks. | EG = DT class sessions involving a preliminary warm-up to music (5-10 min), Tango steps of increasing difficulties, and 5-10 min of free partnered dance  CG = static and dynamic balance exercises, along  with gait training with frequent, unexpected changes of directions while walking along a lane using acoustic cues and exercises for motor coordination | BBS; GDI; TUG; 4SST; 6MWT; FAB; TMT A&B; Stroop Test | DT for PD patients effectively impacts on motor (endurance  and risk of falls) and non-motor functions (executive functions). |
| Dos Santos Delabary et al. (2020)^5^ | (1) PD + dance group (EG)  (2) PD + walking group (CG) | (1) 12  (2) 6 | 24 sessions, each lasting 1 h, twice per week on alternating days | EG= dance program was divided in four parts: joint warm-up, stretching and body sense; strength, balance, and rhythm exercises; exercises with movements thought the room inspired by  samba e do forró basic steps (Brazilian ballroom dance); exploration of movements  in the rhythm of the music; exercises in pairs (couples).  CG= The walking program was performed outdoors on a 400 m | TUG; spatiotemporal gait; parameters of gait | Functional mobility improved similarly in both groups. 12-week program of  Brazilian dance was sufficient to produce improvements in functional mobility and gait in individuals with PD. |
| Hackney et al. (2007)^6^ | (1) PD + tango classes (EG)  (2) PD + exercise classes (CG) | (1) 9  (2) 10 | 2 one-hour sessions per week, for a total of 20 sessions completed within 13 weeks. | EG: tango group participated in progressive tango dance lessons. All training sessions were led by an instructor of American Council on Exercise (ACE)-certified personal trainer.  CG: strength/flexibility exercise classes (adapted from Fit ‘N Fun). | UPDRS, BBS, TUG, Freezing of Gait questionnaire | Both groups showed significant improvements  in UPDRS  score and nonsignificant improvements in self-reported Freezing of Gait.  EG showed significant improvements on the BBS and trend toward improvement on TUG. |
| Hashimoto et al. (2015)^7^ | (1) PD + Dance    (2) PD +exercise    (3) PD + no treatment | (1) 19  (2) 21  (3) 19 | one 60-min session per week, for 12 weeks. | (1) Modern dance methods were used for increasing flexibility, combination of steps, and movements from aerobic, jazz, tango dances and movements from classical ballet.  (2) PD exercises included physical therapy presented by book or video (extending the range of joint motion in upper and lower body, maintaining balance, walking on the spot, rising from and sitting down in a chair, and walking).  (3) no treatment | TUG, BBS, FAB, MRT, AS, SDS, UPDRS | UPDRS significantly improved in the dance group after intervention. Dance was the only intervention to bring about improvement in general symptoms, with a marked effect on mental symptoms in particular. |
| Hulbert et al. (2017)^8^ | (1) PD + dance intervention (EG)  (2) PD + usual care – medications and nursing care (CG) | (1) 15  (2) 12 | 1h, twice/week, for 10 weeks. | EG = partnered dance classes, which covered the basic steps for beginner classes of  ballroom and Latin American dance  CG = usual care | 3-dimensional motion analysis system; Standing Start 180° Turn Test | Those who danced were better able to coordinate their axial and perpendicular segments and  became more ‘en bloc’ in their turning behaviour, suggesting this may be a beneficial adaptation of Parkinson’s |
| Michels et al. (2018)^9^ | (1) PD + dance therapy (EG)  (2) PD + support intervention (CG) | (1) 9  (2) 4 | 60 min weekly over 10 weeks | The DT  sessions emphasized: 1) an understanding of how movement influences  mood and mental health; 2) balance, gait, and coordination; 3) expression  of thoughts, feelings, and emotions through movement and  dance  The  control group practiced no actual physical exercises or techniques. | H&Y; MDS UPDRS; BBS; TUG; PDQ-39; BDI; FSS; VAFS | This pilot study suggests that DT has the potential to improve motor  symptoms in people with PD. Improvement in UPDRS III scores were  seen in DT group although results were non-significant. |
| Kalyani et al. (2020)^10^ | (1) PD + dance group (EG)  (2) PD + individual routine clinical care (CG) | (1) 17  (2) 16 | 1-hour, twice/week for 12 weeks | EG = DfPD® Program: dance classes starting with a 30-minute seated dance, followed by 10-15 minutes each of standing dance with support, then standing dance with movement across the floor. Over time the intervention was progressively advanced to include more complex movements  CG = individual routine clinical care | MDS-UPDRS; TUG; BBS; MBT; TAT; ABC; Gait and Falls questionnaire | DfPD®-based dance classes improved disease-related symptom severity, fine-manual dexterity, and functional mobility. Feasibility of the approach for a large scale RCT was also confirmed. |
| McKee et al. (2013)^11^ | (1) PD + Tango lessons (EG)  (2) PD + Education lessons (CG) | (1) 24  (2) 9 | Twenty 90-minute  Tango or Education lessons over 12 weeks | Adapted Tango: Classes began with practice of previously learned steps and a 20-minute standing warm-up followed by  partnering and rhythmic enhancement exercises. Next, novel step elements were introduced and amalgamated to previously learned steps. | UPDRS-III  BBS  FAB  TUG  4SST | The significant improvement in Tango compared to Education, another highly interactive and  social group activity, suggests motor training inherent to adapted tango could underlie  observed changes. The possibility exists that cognitive gains noted in Tango occurred because aerobic exercise  has beneficial effects upon cognition. |
| Li et al. (2022)^12^ | (1) PD + MMT (EG)  (2) PD + exercise therapy (ETG)  (3) PD + usual medical care and rehabilitation (CG) | (1) 27  (2) 27  (3) 27 | (1) 5 training sessions per week, with each session of 30 min, for 4 weeks  (2) 5 times (1 h each time) every week for 4 weeks.  (3) 5 times (1 h each time) every week for 4 weeks. | EG = Exercises, which were performed to the beat of the music, consisted of flat start walking, turning, narrow space walking, and step  training.  ETG = exercise therapy (i.e. flat walking, turning, narrow space walking, and step training)  without music  CG = conventional treatment including usual medical care and rehabilitation, comprising the following: basic drug treatment; physical factor treatment; and daily life ability training. | 3-dimensional gait analysis system; FOG-questionnaire; UPDRS part II, III | MMT improved gait disorders  in PD patients with FOG, thereby improving their  comprehensive motor function. |
| Thaut et al. (1996)^13^ | (1) RAS (EG)  (2) walking without RAS (CG) | (1) 15  (2) 11 SPT and 11 NT | 3 weeks, walked daily for 30 min for 3 weeks | The RAS program consisted of walking on a  flat surface, stair stepping, and stop-and-go exercises to rhythmically accentuated music at three different tempos. Subjects walked at each tempo for  one third of the exercise time. For the first week of training, the normal tempo  was the pretest cadence, the quick tempo was 5 to  10% faster, and the fast tempo an additional 5 to  10% faster. | -EMG | The improvement was facilitated by nearly equal percentage increases in cadence and stride length. The control group that participated in self-paced training also improved their  gait velocity, but by less than one third of the improvement seen in the RAS group. In addition, some features of EMG  gait-cycle profiles changed toward more normal  muscle-activation patterns. |
| Modugno et al. (2010)^14^ | (1) PD + theater group (EG)  (2) PD + Physiotherapy Rehabilitation (CG) | (1) 10  (2) 10 | EG= 6-h daily sessions, for two consecutive days, once or twice per month, for a total of ~18 h/months, for 3 years  CG= 2- to 3-h daily sessions, 3 days/week (~18 h/month), for 3 years | EG= The initial part of every workshop focused on exercising basic skills.  All subjects were trained in controlling breathing, posture, gait, coordination, and manual tasks. The patients were then taught to approach theater texts and to analyze them. In the second part of the workshop, patients rehearsed singly or in groups, together with actors, based on improvisation or sketches.  Activities of Physiotherapy Rehabilitation  Cardiovascular warm up (10 min)  Stretching exercises (15 min)  Postural exercises (15 min)  Overground gait training (20 min)  Balance training (15 min)  Relaxation exercises (15 min) | UPDRS1; UPDRS2 (ADL); UPDRS3 (motor symptoms); UPDRS4 (complications of the therapy); PDQ39; ESS; HDRS | Active theater has positive and stable effects on the cognitive, affective, and motor domains of PD patients, thus improving their overall QoL |
| Pacchetti et al. (2000)^15^ | (1) PD + MT (EG)  (2) PD + traditional PT (CG) | (1) 16  (2) 16 | EG=13 weekly sessions about 2 hour  CG= weekly sessions, each lasting about 1.5 hours | EG= entrance  and interview, 10 minutes; listening to relaxing music and  visualization of images, 10 minutes; choral singing and facial expression,  breathing, and voice exercises, 15 to 20 minutes; rhythmic  movements, 30  minutes; active music involving collective invention and improvisation,  30 to 40 minutes; free body expression to melodic and rhythmic  music, 20 to 30 minutes; and conversation, 10 minutes.  CG= passive muscle stretching, specific motor tasks and balance training | UPDRS-MS (UPDRS-MS Bradykinesia Factor, UPDRS-MS Rigidity Factor, UPDRS-ADL); PDQL | MT improve motor abilities, emotional status, ADL and quality of life.  PT led to a clear improvement in rigidity but  did not induce any major changes in other variables. |
| Solla et al. (2019)^16^ | (1) PD + dance training (EG)  (2) PD + Conventional care alone (CG) | (1) 10  (2) 10 | (1) Medical therapy plus a 12-week BS dance  Program: The program consisted of 24, 90-min class  sessions, performed twice per week for 12 weeks.  (2) 12-week habitual activities and medical therapy. | BS is typically danced in a closed or open circle by couples who are holding hands, palm to palm. Each 90-min BS session involved three phases. During the initial 30 min, warmup exercises, balance training, coordination, mobilization, ankle control exercises, proprioception, and breathing exercises were performed. During the following 50 min, a Sardinian folk dance teacher conducted the dance supported by traditional records (based on launeddas rhythms). The final 10 min of the 90-min session consisted of deep breathing and static stretching exercises. | - UPDRS-III  -H&Y  -6MWT  -FTSST  -TUG  -BBS  -SRT  -BST | Results of this study indicate BS as a safe and feasible form of physical exercise that is likely to have positive effects on functioning and nonmotor symptoms in IwPD.  Analysis of UPDRS-III scores showed a significant improvement in PD motor symptoms after participation in the BS program. |
| Pohl et al. (2020)^17^ | (1) PD + music-based intervention (EG)  (2) PD + no treatment (CG)  All subjects in Hoehn and Yahr Stages 1-3 | (1) 26  (2) 20 | 2 times per week for 12 weeks. 60 min per session. | (1) Each session was initiated with soft stretching movements and breathing exercises, followed by 50 minutes of exercises typical for the  Ronnie Gardiner Method, and ended with winding down to soft classical music  (2) No treatment | MoCA, TUG (dual task), Test Recall Test, Stroop Color-Word Test, Digit Modalities Test, Mini-BESTest, Falls Efficacy Scale International, Freezing of Gait Questionnaire, PDQ-39 | The study does not support the  efficacy in producing immediate or lasting gains in dual-tasking, cognition, balance, or freezing of gait. Patient-reported outcomes and interviews suggest that the group-based music intervention adds value to mood, alertness, and quality of life in patients with PD |
| Rawson et al. (2019)^18^ | (1) PD+ tango  (2) PD + treadmill  (3) PD + stretching  All subjects in Hoehn and Yahr Stages 1-4 | (1) 43  (2) 41  (3) 35 | 2 times per week for 12 weeks. Class sessions lasted 1 hour. | (1) Argentine tango. Dance partners were spouses, caregivers, volunteers, and laboratory staff.  (2) Participants walked on treadmill at their preferred walking speed. Treadmills were arranged in groups of 4 to allow for social interactions.  (3) Gentle stretching and whole-body flexibility exercises. Social interactions were encouraged. | Forward Velocity, Backward Velocity, SMWT, Mini-BEST Test, MDS-UPDRS-III, PDQ-39 | Not sufficient clinical improvements for all three groups. The maintenance of participation over the course is a critical factor. |
| Duncan et al. (2014)^19^ | (1) PD + argentine tango (EG)  (2) PD + no exercise (CG) | (1) 5  (2) 5 | twice-weekly, 1-hour for 1 year | EG= Argentine tango  CG= participants were given no prescribed  exercise. | MDS-UPDRS (1,2,3); Mini-  BESTest; gait velocity (forward and backward); TUG; dual-task TUG; 6MWT; FOGQ | AT classes improve in motor and non motor symptom severity, subjective ADL performance, and balance. |
| Calabrò et al. (2019)^20^ | (1) PD + RAS treadmill group (EG)  (2) PD + Non RAS (CG) | (1) 25  (2) 25 | (1) DTP + 30 min RAS treadmill group  (2) DTP + 30 min Non RAS | GaitTrainer3 is a platform that integrates gait training via a treadmill and RAS. The device is indeed equipped with an instrumented deck that issues acoustic cues to  determine the exact tempo and rhythm during gait training and visual real-time biofeedback to prompt patients to follow their gait pattern. The  beat frequency was progressively increased up to the target beat frequency (120 bpm) | UPDRS  EEG | Our data suggest that RAS may be a useful, add-on, gait rehabilitation strategy in PD as auditory cueing can specifically target motor cortical beta frequency range synchrony during steady-state treadmill walking in patients  with PD. This modulation sustained greater clinical improvement following RAS gait training than non_RAS gait training. |
| Duncan et al. (2012)^21^ | (1) PD + Argentine Tango group (EG)  (2) no intervention (CG) | (1) 32 (2) 30 | (1) twice weekly, 1-hour Argentine Tango classes for 12 months.  (2) no intervention and no control for attention or socialization. | Argentine Tango (examine the effects of a community-based exercise program on individuals with PD tested off  medication over a 12-month period) | -MDS-UPDRS  -MiniBESTest  - FOG-Q  - 6MWT  - 9HPT | Participants demonstrated a significant reduction in disease severity, as well upper extremity function when compared with controls.  Examination of specific  components of the MDS-UPDRS-3 indicates that tango may  have a positive influence not only on balance and gait, as  might be expected, but also bradykinesia and rigidity |
| Shah et al. (2020)^22^ | (1) PD + PT+MT (EG)  (2) PD + conventional (CG) | (1) 15  (2) 15 | Both of the groups received treatment for duration of 60 minutes,  4 times/week for 6 weeks. | EG = Each session consist of warm up period, workout session and cool down period. Warm period included choral singing and bilateral upper extremity  movement in sitting. Workout session included  passive stretching of major muscle group and strength training; while these exercises, music was played. Cool down  period included breathing exercise with music  and fine motor movement on rocking chair.  CG = conventional PT with almost the same exercise as the EG, but without music | GDI; TUG; PDQ-39 | PT+MT is effective in improving gait, balance and quality of Life in PD patients |
| Shanahan et al. (2017)^23^ | (1) PD + dance therapy (EG)  (2) PD + usual medication treatment (CG) | (1) 20  (2) 21 | The dance group attended a 1.5-hour dancing class each week for 10 weeks and undertook a home dance program for 20 minutes, 3 times per week. The usual care group continued with their usual care and daily activities. | EG = Classes started with a warm-up, targeting movement speed and  size, postural alignment, and other physiological systems required for dance. Exercises were progressed from sitting to  standing according to abilities.  CG = usual medication treatment | UPDRS-motor section; 6MWT; PDQ-39; MBT | Dance therapy may influence quality of life in PD patients |
| Rios Romenets et al. (2015)^24^ | (1) PD + tango (EG)  (2) PD + exercises at home daily (CG) | (1) 18  (2) 15 | Participants attended 1-h  ‘‘traditional Argentine tango’’ classes twice a week for 12 weeks. | Argentine tango intervention included 24  partnered classes. | -UPDRS  -Mini-BESTest  -TUG  -FOGQ | In this randomized controlled trial, we found no clear benefit of Argentine tango upon motor severity of PD. However, among secondary outcomes, we found significant improvement of balance, particularly in dual task TUG, and borderline improvements in cognition and fatigue. |
| Ventura et al. (2016)^25^ | (1) PD + dance therapy (EG)  (2) PD + usual activities (CG) | (1) 8  (2) 7 | 1. 1.25 hours once per week (10 dance classes) 2. Usual activities | EG= 20 minute seated warm-up, 20 minute standing warm-up, dance movements (dance forms included ballet, jazz, Broadway style dance), improvisational movement such as mirroring, or movement across the floor for 20 minutes and concluded in a circle dance in which participants stood facing one another, held hands and passed a “pulse” to the next person in the circle  CG= usual activities | TUG; Gait speed; Standing Balance Test; PDQ-39; PDQ ADL; | The  outcomes with the largest effect sizes included motor function (gait speed),  cognitive switching and QoL. |
| Volpe et al. (2013)^26^ | (1) PD + Irish dance (EG)  (2) PD + physical therapy (CG) | (1) 12  (2) 12 | 1.5 hours  once per week for 6 months. | EG= The protocol incorporated 10 minutes of  warm up range of movement, balance and postural exercises,  70 minutes of Irish dance lessons and a 10 minutes  cool down.  CG= warm  up range of movement and stretching exercises for 10 minutes  followed by 50 minutes of strength training, balance  training and postural re- education, then 20 minutes of  gait training and a 10 minute cool down. | UPDRS3 (motor section  Scores); TUG; BBS; FOG; PDQ39 | Irish set dancing  improve mobility, reduce  disability and enhance health related quality of life. |
| Naro et al. (2023)^27^ | (1) PD + RAS (EG)  (2) PD (CG) | (1) 25  (2) 25 | (1) RAS daily 30-minute 5 days weekly, for two months.  ALL patients received in addition a daily training program that included 45 minutes of traditional overground gait training, 45 minutes of activities of daily living and reaching activities in occupational therapy, 45 minutes of biomechanical training in both the upper and lower limbs, 30 minutes of speech therapy, and 30 minutes of rest distributed between sessions (for a total of 195 minutes). | EG = session of RAS during treadmill gait training using the Gait-Trainer3  CG = an equal amount of gait training without using RAS. | FGA, UPDRS, BBS, FES, 10MWT, TUG, and GQI as clinical outcome measures and EEG data | Both trainings yielded a significant improvement in FES, FGA, and UPDRS; the changes were of greater magnitude in the RAS group than in the non-RAS group concerning FGA and UPDRS. Conversely, the groups equally improved in FES, BBS, and TUG. The 10MWT only slightly improved in both groups |
| Raglio et al. (2023)^28^ | (1) PD + gait training program supported by sonification (EG)  (2) PD + gait training program without sonification (CG) | (1) 10  (2) 9 | 20 sessions, 30 minutes, thrice-weekly, of the same gait training  program  -first phase (15 min),  warm-up with therapeutic exercises for gait re-education by dividing the walking motor sequence  into different phases and progressively re-educating them  - second phase (also 15 min, 7 min of walking, 1 min rest, 7 min of walking). The patient is asked to slightly increase the pace of their steps to the maximum  possible speed | EG = sonification is a predefined sound sample is played at  specific moments of the movement. A chord progression with a click on each musical beat guides exercise speed | FIM (cognitive and motor)  6MWT, MiniBESTest, Dynamic Gait Index, TUG, VAS, McGill QoL, GPE. | EG reported being more fatigated than the CG.  Balance, measured with Mini-BESTtest and DGI, improved significantly in the EG and not in the CG.  There were no significant results in other secondary outcome measures |
| Rabini et al. (2024)^29^ | (1) PD + Argentine Tango (EG)  (2) PD + physiotherapy (CG)  All subjects in Hoehn and Yahr Stages 2-3 | (1) 12  (2) 12 | 4-month group intervention 1 h, twice a week, for  a total of 32 h | EG = partners were healthy volunteers, who were generally not experts in tango dance. Participant in this activity danced in both the leading  and the following role  CG = mobility, balance, muscle strengthening exercises and also for cardiovascular health. Each week the difficulty increased | Mini-BESTest, Berg Balance Scale, Four Step Square (FSS), TUG, 10-m walking test—fast condition, 6MWT, 30 s sit-to-stand, 5 time sit-to-stand (5STS), 10-m walking test—slow condition, 9-hole peg test, FaSS, FGT, ABC, FES e MDS-UPDRS-III | Physiotherapy revealed significant differences on miniBESTest. The two groups improved from PRE measures to POST but no significant differences were identified. |

ABC = Activities-specific balance confidence scale; ADL = Activities of Daily Living; AES-C = Apathy Evaluation Scale-clinician version; AS = Apathy Scale; BBS = Berg Balance Scale; BBT = Box and Block Test; BDI-II = Beck Depression Inventory-II; Brief-COPE = Brief Coping Orientation to Problems Experienced; BS = Ballu Sardu - folk dance; BST = Back Scratch Test; CG = Control Group; DfPD® = dance program designed for people with PD; DT = Dance Therapy; EG = Experimental Group; ESS = Epworth Sleepiness Scale; ETG = Exercise Therapy Group; FAB = Fullerton Advanced Balance Scale; FaSS = Fatigue Severity Scale; FES = Falls Efficacy Scale; FGA = Functional Gait Assessment; FGT = Freezing of Gate Test; FIM = Functional Independence Measure; FOG = Freezing of Gait; FOGQ = Freezing of Gait Questionnaire; FSS = Fatigue Severity Scale; FTSST = Five Times Sit-to-Stand Test; GDI = Gait Dynamic Index; GPE = Global Perceived Effect; GQI = Gait Quality Index; HDRS = Hamilton Depression Rating Scale; H&Y = Hoen & Yahr; MBT = Mini-BESTest; MDS-UPDRS = Movement Disorder Society - Unified Parkinson's Disease Rating Scale; MMT = Music-based movement therapy; MoCA = Montreal Cognitive Assessment; MRT = Mental Rotation Task; MT = Music Therapy; NMT = Neurologic Music Therapy; NT = no-training group; PD = Parkinson’s Disease; PDQ-39 = Parkinson's Disease Questionnaire; PDQL = Parkinson’s Disease Quality of Life Questionnaire; PGWBI = Psychological General Well-Being Index; PPT = Purdue Pegboard Test; PSE = Pattern Sensory Enhancement; PT = physical therapy; Q-DASH = Quick Disability of Arm, Shoulder, and Hand; QoL = Quality of Life; RAS = Rhythmic Auditory Stimulation; SDS = Self-rating Depression Scale; SMWT = Six-Minute Walk Test; SPT = self internally-paced group; SRT = Sit-and-Reach Test; TAT = Tinetti Assessment Tool; TIMP = Therapeutic Instrumental Music Performance; TMT A&B = Trail Making Test part A&B; TR = Traditional Rehabilitation; TUG = Time Up and Go; VAFS = Visual Analog Fatigue Scale; 4SST = 4 Square-Step Test; 6MWT = 6 Minute Walking Test; 9HPT = 9-Hole Peg Test; 10mWT = 10 meter Walking Test.

| Table 2S. Risk of bias in non-randomized studies | | | | | | | | |
| --- | --- | --- | --- | --- | --- | --- | --- | --- |
| **Study name** | **Confounding bias** | **Selection bias** | **Measure intervention bias** | **Performance bias** | **Attrition bias** | **Detection bias** | **Reporting bias** | **Overall bias** |
| Dos Santos Delabary et al., 2020^5^ | **Low risk**  Each variable that could potentially influence the behavior of the results was tested separately as a covariate in each model. | **Low risk**  All eligible participants included in the study; participant's start of follow up and start of intervention coincided. | **Moderate risk**  Intervention status is well defined; Intervention definition is based solely on information collected at the time of intervention. No info on whether the interventions status could have been affected by knowledge of the outcome or risk of the outcome. | **Low risk**  Deviations from usual practice were not detected | **Low risk**  Outcome data fully presented | **Low risk**  Methods of outcome assessment were comparable across intervention groups and outcome measures could not have been influenced by knowledge of the intervention received (i.e., TUG). | **Serious risk**  PDQ-39 and UPDRS III present in the protocol but not reported in the study | **Serious risk**  Serious risk of the presence of bias related to the selection of reported results. |
| Ventura et al., 2016^25^ | **Serious risk**  Confounding expected, all known important confounding domains not measured and controlled for. | **Moderate risk**  All eligible participants included in the study; participant's start of follow up and start of intervention probably coincided. | **Moderate risk**  Intervention status is well defined; No info on major aspects of the assignments of intervention status | **Low risk**  Deviations from usual practice were not detected | **Low risk**  Outcome data fully presented | **Low risk**  Methods of outcome assessment were comparable across intervention groups; The outcome was assessed by assessors not aware of the intervention received by study participants | **No information**  No information on the presence of a protocol or a statistical analysis plan. | **Serious risk**  Serious risk for the presence of bias related to confounding factors not measured and controlled for. No information on the presence of a protocol to detect reporting bias. |

**Tables 1S and 2S References**

1. Bastepe-Gray S, Wainwright L, Lanham DC, et al. GuitarPD: A Randomized Pilot Study on the Impact of Nontraditional Guitar Instruction on Functional Movement and Well-Being in Parkinson's Disease. *Parkinson's disease.* 2022;2022.

2. Bukowska AA, Krężałek P, Mirek E, Bujas P, Marchewka A. Neurologic music therapy training for mobility and stability rehabilitation with parkinson’s disease - A pilot study. *Frontiers in human neuroscience.* 2016;9(JAN2016).

3. De Luca R, Latella D, Maggio MG, et al. Do patients with PD benefit from music assisted therapy plus treadmill-based gait training? An exploratory study focused on behavioral outcomes. *The International journal of neuroscience.* 2020;130(9):933-940.

4. De Natale ER, Paulus KS, Aiello E, et al. Dance therapy improves motor and cognitive functions in patients with Parkinson's disease. *NeuroRehabilitation.* 2017;40(1):141-144.

5. Dos Santos Delabary M, Monteiro EP, Donida RG, Wolffenbuttel M, Peyré-Tartaruga LA, Haas AN. Can Samba and Forró Brazilian rhythmic dance be more effective than walking in improving functional mobility and spatiotemporal gait parameters in patients with Parkinson's disease? *BMC neurology.* 2020;20(1):305.

6. Hackney ME, Kantorovich S, Levin R, Earhart GM. Effects of tango on functional mobility in Parkinson's disease: a preliminary study. *Journal of neurologic physical therapy : JNPT.* 2007;31(4):173-179.

7. Hashimoto H, Takabatake S, Miyaguchi H, Nakanishi H, Naitou Y. Effects of dance on motor functions, cognitive functions, and mental symptoms of Parkinson's disease: a quasi-randomized pilot trial. *Complementary therapies in medicine.* 2015;23(2):210-219.

8. Hulbert S, Ashburn A, Roberts L, Verheyden G. Dance for Parkinson's-The effects on whole body co-ordination during turning around. *Complementary therapies in medicine.* 2017;32:91-97.

9. Michels K, Dubaz O, Hornthal E, Bega D. “Dance Therapy” as a psychotherapeutic movement intervention in Parkinson's disease. *Complementary therapies in medicine.* 2018;40:248-252.

10. Kalyani HH, Sullivan KA, Moyle GM, Brauer SG, Jeffrey ER, Kerr GK. Dance improves symptoms, functional mobility and fine manual dexterity in people with Parkinson disease: a quasi-experimental controlled efficacy study. *European journal of physical and rehabilitation medicine.* 2020;56(5):563-574.

11. McKee KE, Hackney ME. The effects of adapted tango on spatial cognition and disease severity in Parkinson's disease. *Journal of motor behavior.* 2013;45(6):519-529.

12. Li KP, Zhang ZQ, Zhou ZL, et al. Effect of music-based movement therapy on the freezing of gait in patients with Parkinson’s disease: A randomized controlled trial. *Frontiers in aging neuroscience.* 2022;14.

13. Thaut MH, McIntosh GC, Rice RR, Miller RA, Rathbun J, Brault JM. Rhythmic auditory stimulation in gait training for Parkinson's disease patients. *Movement disorders : official journal of the Movement Disorder Society.* 1996;11(2):193-200.

14. Modugno N, Iaconelli S, Fiorilli M, Lena F, Kusch I, Mirabella G. Active theater as a complementary therapy for Parkinson's disease rehabilitation: A pilot study. *TheScientificWorldJournal.* 2010;10:2301-2313.

15. Pacchetti C, Mancini F, Aglieri R, Fundaró C, Martignoni E, Nappi G. Active music therapy in Parkinson's disease: An integrative method for motor and emotional rehabilitation. *Psychosomatic medicine.* 2000;62(3):386-393.

16. Solla P, Cugusi L, Bertoli M, et al. Sardinian Folk Dance for Individuals with Parkinson's Disease: A Randomized Controlled Pilot Trial. *Journal of alternative and complementary medicine (New York, NY).* 2019;25(3):305-316.

17. Pohl P, Wressle E, Lundin F, Enthoven P, Dizdar N. Group-based music intervention in Parkinson's disease - findings from a mixed-methods study. *Clinical rehabilitation.* 2020;34(4):533-544.

18. Rawson KS, McNeely ME, Duncan RP, Pickett KA, Perlmutter JS, Earhart GM. Exercise and Parkinson Disease: Comparing Tango, Treadmill, and Stretching. *Journal of neurologic physical therapy : JNPT.* 2019;43(1):26-32.

19. Duncan RP, Earhart GM. Are the effects of community-based dance on Parkinson disease severity, balance, and functional mobility reduced with time? A 2-year prospective pilot study. *Journal of Alternative and Complementary Medicine.* 2014;20(10):757-763.

20. Calabrò RS, Naro A, Filoni S, et al. Walking to your right music: A randomized controlled trial on the novel use of treadmill plus music in Parkinson's disease. *Journal of NeuroEngineering and Rehabilitation.* 2019;16(1).

21. Duncan RP, Earhart GM. Randomized controlled trial of community-based dancing to modify disease progression in Parkinson disease. *Neurorehabilitation and neural repair.* 2012;26(2):132-143.

22. Shah V, Yeole U, Purandare SS, Vishwakarma KR. Effect of physical therapy with music therapy on gait, balance and quality of life in parkinson’s disease. *Indian Journal of Public Health Research and Development.* 2020;11(6):1075-1080.

23. Shanahan J, Morris ME, Bhriain ON, Volpe D, Lynch T, Clifford AM. Dancing for Parkinson Disease: A Randomized Trial of Irish Set Dancing Compared With Usual Care. *Archives of physical medicine and rehabilitation.* 2017;98(9):1744-1751.

24. Rios Romenets S, Anang J, Fereshtehnejad SM, Pelletier A, Postuma R. Tango for treatment of motor and non-motor manifestations in Parkinson's disease: A randomized control study. *Complementary therapies in medicine.* 2015;23(2):175-184.

25. Ventura MI, Barnes DE, Ross JM, Lanni KE, Sigvardt KA, Disbrow EA. A pilot study to evaluate multi-dimensional effects of dance for people with Parkinson's disease. *Contemporary clinical trials.* 2016;51:50-55.

26. Volpe D, Signorini M, Marchetto A, Lynch T, Morris ME. A comparison of Irish set dancing and exercises for people with Parkinson's disease: a phase II feasibility study. *BMC geriatrics.* 2013;13:54.

27. Naro A, Pignolo L, Bruschetta D, Calabrò RS. Data on a novel approach examining the role of the cerebellum in gait performance improvement in patients with Parkinson disease receiving neurologic music therapy. *Data in brief.* 2023;47:109013.

28. Raglio A, De Maria B, Parati M, et al. Movement Sonification Techniques to Improve Balance in Parkinson's Disease: A Pilot Randomized Controlled Trial. *Brain sciences.* 2023;13(11).

29. Rabini G, Meli C, Prodomi G, et al. Tango and physiotherapy interventions in Parkinson's disease: a pilot study on efficacy outcomes on motor and cognitive skills. *Scientific reports.* 2024;14(1):11855.
